# Five-year follow-up results of the Italian case–control study comparing poem and laparoscopic Heller myotomy using the propensity score

**DOI:** 10.1007/s00464-026-12910-6

**Published:** 2026-05-21

**Authors:** Andrea Costantini, Francesca Mangiola, Giovanni Capovilla, Rosario Landi, Luca Provenzano, Loredana Nicoletti, Michele Valmasoni, Cristiano Spada, Mario Costantini, Pietro Familiari, Renato Salvador

**Affiliations:** 1 Department of Surgical, Oncological and Gastroenterological Sciences, University of Padua, Padua, Italy; 2 Digestive Endoscopy Unit, Fondazione Policlinico Agostino Gemelli IRCCS, Università Cattolica del Sacro Cuore, Rome, Italy

**Keywords:** Achalasia, Follow-up, LHD, POEM, Propensity score matching

## Abstract

**Background:**

All studies in the literature comparing POEM and laparoscopic Heller-Dor fundoplication (LHD) for achalasia are limited by a short follow-up. The aim of the study was to reassess the patients of our study at a minimum 5-year follow-up.

**Methods:**

Two groups of consecutive patients undergoing treatment for primary achalasia between 2014 and 2017 were recruited in two high-volume centers, one with extensive experience with POEM and one with LHD, and were matched with a propensity score. Only those patients who reached a minimum 5-year follow-up were considered in this study.

**Results:**

A total of 68 patients in the POEM group and 118 in the LHD formed the study population. One patient in each group died during follow-up for causes unrelated to achalasia. At a median follow-up of 79.6 months (IQR 42.1–103.95), 87.3% of the POEM patients and 90.1% of the LHD patients showed an Eckardt score ≤ 3 (*p* = n.s.). The need for PPIs (45.6% vs 24.6%, *p* = 0.03) and the rate of esophagitis (34% vs 10.4%, *p* < 0.002) were higher in the POEM group. None of the patients developed esophageal cancer. The disease-free curve estimates at 1–3–5 years were similar in the 2 groups (99–98–96% vs 99–96–96%, *p* = 0.50). At a minimum 5-year follow-up, only 9 patients in the POEM group and 12 patients in the LHD group had symptomatic recurrence.

**Conclusion:**

POEM provides the same long-term results as LHD. This study confirms, however, a higher incidence of postoperative GERD with the former, even if its real significance needs to be further evaluated.

Esophageal achalasia (EA) is characterized by the impaired relaxation of the lower esophageal sphincter (LES) and the absence of esophageal peristalsis; its pathogenesis is still unknown [1]. As a result, although no definitive therapy is available, an effective and durable palliation of symptoms has been achieved in most patients by disrupting the LES muscle fibers with forceful endoscopic pneumatic dilations (PD) or by dividing them by means of a laparoscopic Heller’s myotomy (LHM) [2], that in the past 3 decades represented the gold standard treatment. This has been challenged in the last 15 years, after the introduction of a new endoscopic procedure, the so-called per-oral endoscopic myotomy (POEM) [3]. This was rapidly accepted throughout the world, achieving good short- to mid-term results and imposing itself as a candidate for replacing LHM (and PD, as well) as the first-line therapy for EA [4–12]. This was based on several initial retrospective studies reporting excellent outcomes of the procedure, and some meta-analysis, comparing POEM to LHM. Finally, about 5 years ago, a European randomized control trial (RCT) [13] and a case–control study using the Propensity Score Matching (PSM), both comparing POEM and LHM, were published (the latter by our own group) [14]: two years after the procedure, POEM was non-inferior to LHM in terms of efficacy even if a concern about reflux after POEM was consistently raised.

The aim of the present study was to reevaluate (at 5 years follow-up) the population previously analyzed in our PSM case–control study, and to compare the long-term outcome of POEM and laparoscopic Heller-Dor (LHD) for the treatment of EA.

## Materials and methods

### Patients

This was a multicenter, case–control study in which all consecutive patients with a diagnosis of EA were enrolled between 2014 and 2017 and had undergone either POEM or LHD in two high-volume Italian institutions with extensive experience in the two procedures: the Digestive Endoscopy Unit of the Fondazione Policlinico Universitario Agostino Gemelli IRCCS in Rome for POEM [15] and the Department of Surgical, Oncological and Gastroenterological Sciences of the University of Padua for LHD [16]. All the data regarding the patient’s enrollment, the preoperative assessment, the treatment and the follow-up protocol have already been reported in a previous paper [14]. Patients who had already undergone surgery or POEM for EA were ruled out, as well as patients belonging to the so-called learning curve (set at the first 20 cases for each single operator [16, 17]). Also, some patients who had undergone the procedure during some “teaching” courses or master classes, with missing data or eventually diagnosed as having a motor disorder different from EA, were excluded. On the contrary, patients who had received unsuccessful “traditional” endoscopic treatment (i.e., PD and/or Botox injections) were included in the study. The study protocol is reported in Table 1.

**Table 1:** Summary of the study protocol for the two groups of patients. (# = POEM group only)

Procedure	Pre-treatment	2 months	6 months	1 year	Every 2 years
Symptom assessment	●	●	●	●	●
Barium swallow	●	●			
Endoscopy	●		#	●	●
HRM	●		●		
pH-monitoring			●*		

^*^pH-monitoring performed off-acid suppression

The study was approved by the Research Committee of the Department of Surgical, Oncological, and Gastroenterological Sciences – University of Padova. All the patients of both Centers signed informed consent to have their data recorded in a dedicated database. The study was conducted in accordance with the ethical principles of the Declaration of Helsinki [18].

### Follow-up and outcome

The 5-year follow-up was achieved by means of a recent endoscopy and a clinical evaluation. If patients did not attend the outpatient clinic, they were evaluated with a telephonic interview and the evaluation of results (via email) of a recent endoscopy. Treatment failure was defined as the persistence or recurrence of an Eckardt score (ES) > 3 or the need for retreatment, based on the comprehensive evaluation of the diagnostic tools previously described.

### Statistical analysis

As explained in a previous work [14], for the best possible matching of the two groups of patients, we used the PSM, a statistical method that attempts to reconstruct a situation similar to randomization [19]. For the calculation of the PSM, the following variables were considered: age, sex, duration of symptoms, previous endoscopic treatment(s), Eckardt score, radiological stage, and manometric pattern at HRM. We selected these variables since they may affect the outcome of the different achalasia treatments, as reported in a number of previous studies [1, 16, 20]. For the best possible pairing of the patients of the two groups, a caliper of 0.2 of the standard deviation of the PSM logit was used [21].

The data were expressed as medians and interquartile ranges (IQR) for continuous variables and as counts or proportions (%) for categorical variables. Non-parametric tests were used to compare groups (Mann–Whitney and Wilcoxon, as appropriate). Fisher’s exact test was used to compare categorical data. Symptom-control survival estimates were calculated with the Kaplan–Meier method and survival comparisons were performed using the log-rank test. A probability of 5% was assumed to be statistically significant (*p* = 0.05). The statistical analyses were performed by means of “R” statistical software [22].

## Results

As reported in a previous paper [14], during the enrollment period, 318 consecutive patients underwent POEM at the Digestive Endoscopy Unit of the Fondazione Policlinico Universitario Agostino Gemelli IRCCS, Rome, Italy. During the same period, 242 patients received LHD at the Department of Surgical, Oncological and Gastroenterological Sciences of the University of Padua, Italy.

The calculation of the PSM with a caliper of 0.233 allowed the selection of 140 pairs of strictly matched patients who represent the study population. Table 2 summarizes the demographic and preoperative characteristics of the two groups of patients: no statistically significant differences were found, both in the parameters considered for the PSM and in all the others.

**Table 2:** Demographic and clinical characteristics of the two groups of patients: no statistically significant differences were found, both in the parameters considered for the PS and in all the other. Data are expressed as Median and IQR

	POEM (*n* = 140)	LHD (*n* = 140)	*p* value
*Sex (M:F)	70:70	73:67	1
*Age (years)	47 (38–60)	48 (38–59)	0.90
Weight (kg)	66 (57–77)	65 (55–73)	0.20
Height (cm)	169 (160.5–176)	169 (162–176)	0.79
BMI (kg/m^2^)	23 (20.3–26.2)	22 (19.4–21.7)	0.43
*Symptom duration (months)	24 (12–39)	24 (12–60)	0.49
*Eckardt’s score	7.5 (5.75–9)	8 (5.75–9)	0.35
Esophageal diameter (cm)	4.4 (3.25–5.35)	4.5 (3.5–5.0)	0.67
*Previous endoscopic treatment	9.3%	10%	0.35
*Radiological stage			
Stage I	29 (20.7%)	31 (22.1%)	0.63
Stage II	78 (55.7%)	83 (59.3%)	0.44
Stage III	24 (17.2%)	19 (13.6%)	0.88
Stage IV	9 (6.4%)	7 (5.0%)	0.64
*Manometric type			
Type I	28 (20.0%)	35 (25.0%)	0.07
Type II	94 (67.2%)	89 (63.6%)	0.83
Type III	10 (7.1%)	13 (9.1%)	0.64
n/a	8 (5.7%)	3 (2.2%)	0.12
LES basal pressure (mmHg)	41 (29–52)	42 (32–60)	0.13
4sIRP1 (mmHg)	26 (20–35)	31 (23–43)	0.91

1 4sIRP = 4-s integrated relaxation pressure, calculated as the average of minimum relaxation pressure for 4 s after swallowing

* = parameters considered for the calculation of the PS

Data regarding the operative time, the postoperative stay, the adverse events [21] were described in the previous paper [14] and did not differ between the two groups. Complications accounted for 5% in the POEM group and 2.1% in the LHD group, being mostly mucosal perforations repaired with clips during POEM and sutured during laparoscopy. One patient in each group died at 3 and 24 months, respectively, in the POEM and LHD groups, for causes unrelated to the disease or the performed procedure (natural causes and prostatic cancer, respectively).

### Symptomatic outcome

**Fig. 1:**
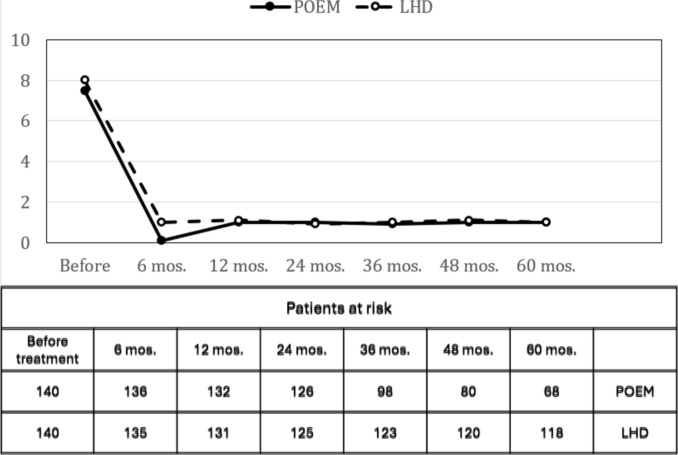
Variation of the Eckardt score at the different follow-up evaluations after POEM (solid gray) and LHD (white columns). The number of evaluated patients at the different points is also shown

A minimum follow-up of 5 years was achieved in 68 patients in the POEM group and in 118 patients in the LHD group, with a median follow-up of 85 months (IQR 65–103) and 101 months (IQR 65–103), respectively. The median postoperative ES did not differ in the two groups, being 1 (IQR 0–2) in both (p 0.19). Moreover, 61 out of 68 patients in the POEM group (89.7%) and 106 out of 118 patients in the LHD group (89.8%) had an ES ≤ 3 (p 0.98) and, therefore, were considered to have a successful treatment. Figure 1 represents the variation of the median ES in both groups, as evaluated at different follow-up points.

**Fig. 2:**
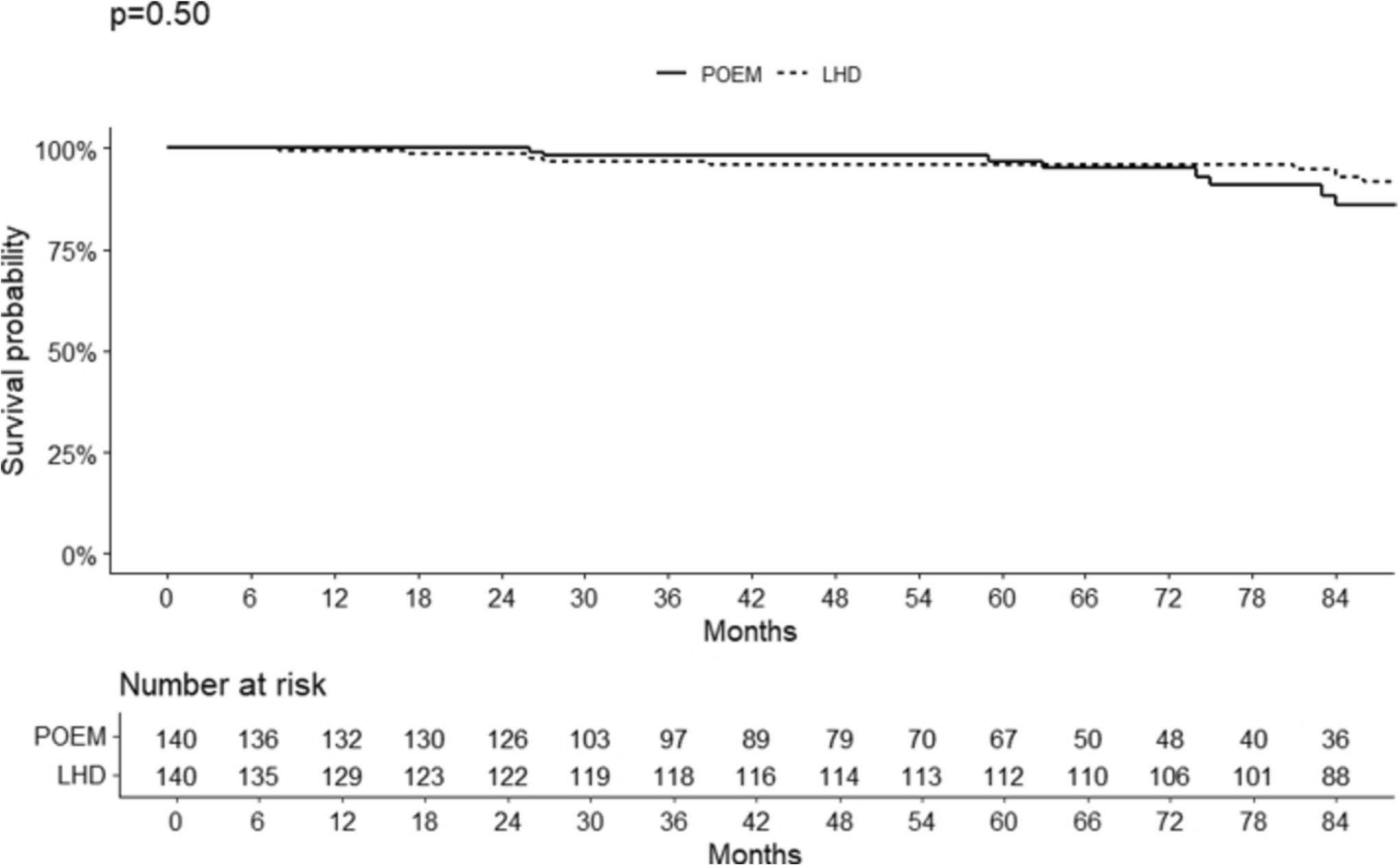
Kaplan–Meier survival curves for both methods showed a similar pattern, without significant differences between the two groups. In particular, nearly 7 years after the operation, the probability of having good control of achalasia symptoms was higher than 90% for both treatments (POEM 98.2%, LHD 93.9%, *p* = 0.2, log-rank test)

In Fig. 2, the symptom-control survival curves are illustrated: the two curves show a similar trend, without significant differences between the two groups. Nearly 7 years after the operation, the probability of having EA symptoms controlled was about 90% in both treatment groups (POEM 87.3%, LHD 90.1%, *p* 0.5, log-rank test). Failure was detected in 7 patients in the POEM group and 12 patients in the LHD group (p 0.19). Symptoms recurred 22 months after LHD (IQR 10–84) and were treated with a median of 2 complementary PD (IQR 1–3), with resolution of recurrent symptoms, whereas of the 7 patients in the POEM group, one underwent PD and 5 had clinical palliation of their symptoms with PPI. One last patient required LHD, with complete resolution of symptoms.

### Function evaluation

Most of the patients who had a 5-year follow-up accepted to undergo esophageal tests. In particular: 53 out of 68 patients in the POEM group (77.9%) had postoperative endoscopy, whereas 54 (79.4%) had 24-h pH monitoring. In the LHD group, 106 out of 118 patients (89.8%) had endoscopy and 88 (74.6%) had a 24-h pH-monitoring of the distal esophagus.

Function tests showed, 6 months after the endoscopic or surgical treatment, a statistically significant difference in the postoperative exposure of the distal esophagus to acid in the two groups (Table 3). Moreover, the acid exposure time % (AET%) was 2.8% (IQR 0.2–26.5) in the patients who received POEM, as compared to 0.3% (IQR 0–7.7) of the patients who had LHD, and the median DeMeester’s score was 12.2 (IQR 1.3–115.7) and 1.5 (IQR 0–35.1), respectively (*p* < 0.01 in both cases). By evaluating the single patients, 16 out of the 54 patients evaluated after POEM (29.6%) showed an abnormal exposure of the distal esophagus to acid, as compared to only 8 of the 88 patients evaluated after LHD (9.1%) (*p* < 0.01).

**Table 3:** Functional and endoscopic parameters after the two procedures

Parameter	POEM (*n* = 68)	LHD (*n* = 118)	*p* value
Follow-up (months)	85 (65–103)	101 (87–110)	** < 0.01**
Eckardt score	1 (0–2)	1 (0–2)	0.19
Success	61 (89.7%)	106 (89.8%)	0.98
Failure	7 (10.3%)	12 (10.2%)	
PPI			
Yes	33 (48.5%)	28 (24.6%)	** < 0.01**
No	35 (51.5%)	86 (75.4%)	
Esophagitis			
Yes	18 (34%)	11 (10.4%)	** < 0.01**
No	35 (66%)	95 (89.6%)	
Esophagitis severity			
A	12 (22.6%)	7 (6.6%)	0.87
B	6 (11.3%)	3 (2.8%)	
C	0	1 0.9%)	
D	0	0	
Median AET	2.8 (0.2–26.5)	0.3 (0.0–7.7)	** < 0.01**
Positive pH-monitoring (Lyon 2.0)			
Yes	16 (29.6%)	8 (9.1%)	** < 0.01**
No	38 (70.4%)	80 (90.9%)	
AET < 6%	1.4 (0.2–4.2)	0.7 (0.0–2.8)	** < 0.01**
AET ≥ 6%	15.0 (5.4–37.2)	8.6 (6.4–18.1)	.62
DeMeester score	12.2 (1.3–112.6)	1.5 (0.0–35.1)	** < 0.01**

Bold values indicate statistical significance (*p* < 0.05)

Also, the prevalence of endoscopic esophagitis was, after treatment, significantly higher in the POEM group than in the LHD group: endoscopic esophagitis of any degree was found in 34% of patients of the former, as compared to 10.4% of patients of the latter group (*p* < 0.01) (Table 3). However, it must be underlined that the majority of esophagitis belonged to grade A: more severe forms of esophagitis (grade B–D of the Los Angeles classification) were detected only in a minority of patients, and no statistically significant difference was found (11.3% and 3.9%, respectively, *p* 0.06), even though the low number of cases may lead to a possible type II (or beta) error.

Finally, the patients with a good outcome (Table 4) and those with failure (Table 5) after each procedure were compared. In the first case, a higher PPI use, and a higher incidence of esophagitis and pathological pH-monitoring evaluations were found in the POEM group than in the LHD group (*p* < 0.01). However, only pH parameters were found to be different between the patients who failed their procedure, being (again) higher in the failed POEM group.

**Table 4:** Functional and endoscopic parameters of the success groups of both procedures

Parameter	Successful POEM (*n* = 61)	Successful LHD (*n* = 106)	*p*-value
Follow-up (months)	87 (64–105)	103 (87–110)	** < 0.01**
Eckardt score	1 (0–2)	1 (0–1)	0.14
PPI			
Yes	29 (47.5%)	25 (24.3%)	** < 0.01**
No	32 (52.5%)	78 (75.7%)	
Esophagitis			
Yes	17 (36.2%)	9 (9.2%)	** < 0.01**
No	30 (63.8%)	89 (90.8%)	
Esophagitis severity			
A	12 (25.5%)	5 (5.1%)	0.67
B	5 (10,6%)	3 (3.1%)	
C	0	1 (1%)	
D	0	0	
Median AET	2.7 (0.2–19.5)	0.3 (0.0–8.1)	** < 0.01**
Positive pH-monitoring (Lyon 2.0)			
Yes	14 (28.6%)	8 (10.1%)	**0.01**
No	35 (71.4%)	71 (89.9%)	
DeMeester score	11.7 (1.2–95)	1.7 (0.0–37.8)	** < 0.01**

Bold values indicate statistical significance (*p* < 0.05)

**Table 5:** Functional and endoscopic parameters of the failure groups of both procedures

Parameter	Failed POEM (*n* = 7)	Failed LHD (*n* = 12)	*p*-value
Follow-up (months)	83 (75–93)	93 (89–102)	0.09
Eckardt score	5 (4–7)	4 (4–5)	0.35
PPI			
Yes	4 (57.1%)	3 (25%)	0.33
No	3 (42.9%)	9 (75%)	
Esophagitis			
Yes	1 (16.7%)	2 (25%)	1
No	5 (83.3%)	6 (75%)	
Esophagitis severity			
A	0	2 (25%)	0.33
B	1 (16.7%)	0	
C	0	0	
D	0	0	
Median AET	4.2 (1.5–9.6)	0 (0–1)	**0.02**
Positive pH-monitoring (Lyon 2.0)			
Yes	2 (40%)	0	0.11
No	3 (60%)	9 (100%)	
DeMeester score	15.4 (6.3–44.7)	0.7 (0.5–2.9)	**0.03**

Bold values indicate statistical significance (*p* < 0.05)

## Discussion

In the past decades, LHD has represented the treatment of choice for EA in many centers, being generally preferred to PD that provided similar long-term results but with an “on demand” repetitive protocol only [23, 24]. Fifteen years ago, POEM was introduced [3], rapidly spreading among physicians as an attractive alternative treatment for EA, even though no proper comparative studies with other consolidated techniques (namely LHD) were reported. Initial reports with short- and medium-term results of this technique were extremely good, at least equal to those of LHD. A number of retrospective studies [6, 25–29] and meta-analyses [8–12], comparing the new technique with the laparoscopic myotomy, soon appeared in the literature, but it was only at the end of 2019 (ten years after the introduction of POEM) that a European RCT comparing POEM and LHD was published [13], showing similar outcomes with the two techniques: at a 2-year follow-up, POEM was non-inferior to LHD in terms of efficacy (clinical success rate). POEM, however, resulted in more cases of post-procedural GERD in terms of the rate of esophagitis and the PPI use, a *caveat* that confirmed observations on early retrospective case series. In the same period, a paper by our group, comparing two similar groups of patients selected by a PSM-based case–control study, was published [14], showing exactly similar results of the RCT study: very good control of symptoms and safety profile with both techniques. This study also showed a higher incidence of post-procedural GERD with POEM. However, both these studies reported a too short follow-up (2 years) for a chronic disease such as EA.

The results of the present follow-up study of the previously published paper confirmed, at a minimum 5-year follow-up, that LHD still represents a very efficient technique for the treatment of EA, and that the newcomer POEM is not inferior to the former, both offering long-term symptom-relief in the region of 90%. These results are in line with those reported in the literature for each method singularly evaluated [29] and, above all, with the results of the 5-year follow-up of the European RCT also recently published [30], where symptom control was obtained in 75% and 70.8%, respectively, for POEM and LHD. One aspect in which our study differs from this RCT is the higher success rate we reported at a minimum 5-year follow-up: 89.7% for POEM and 89.8% for LHD. This may be related to the different typology of the two studies (RCT vs retrospective with PSM, albeit the latter is considered the best approximation to the former, being considered a “quasi-randomization” method) [18]. In our opinion, the differences in outcomes were more likely related to the multicentric characteristic of the RCT, needed to reach good sample sizes due to the rarity of the disease, and in which the expertise with one or the other technique could not have been equally high among all the participant centers. In our study, the two groups of patients were treated with the two different techniques in two different Italian centers, with the highest expertise with POEM or, respectively, LHD, thus receiving the best possible operation. This surely does not represent the real-world situation and may represent an important limit of our study. Other authors [31] also used PSM to match patients who underwent POEM or LHM at their institution. The number of POEM patients, however, was small as compared to the patients who underwent LHM (88), with a resulting matching of 1:3 for the two groups. Moreover, the time span in which the two groups of patients were recruited was very different (2014–2015 for POEM and 2005–2015 for LHM) and no mention was made of patients treated during the learning curve. Despite these limitations, however, their results were remarkably like those of the present study, adding further strength to our findings.

Even at a 5-year follow-up, our study further confirmed the finding, already known, of a higher incidence of postprocedural GERD after POEM compared to LHD, with a consequently higher postoperative use of PPIs after POEM than after LHD: about half the patients who underwent POEM were taking PPIs, as compared to one-quarter of the patients who underwent LHD. This reflects also the higher percentage of postprocedural esophagitis we found at the last endoscopy in the POEM group compared to the LHD; however, the majority of these cases of esophagitis belonged to grade A of the Los Angeles classification, and when considering esophagitis of grade B or more, this difference fades away, probably reflecting a type-2 error for the little numerosity of patients with grade B or higher esophagitis in both groups. The already quoted 5-year follow-up paper of the European RCT reports similar results. [30]. Several other retrospective studies had already reported this finding [15, 32, 33]. Of course, GERD is a problem that must be considered when treating EA. It is however a problem whose relevance is perhaps overemphasized and whose consequences are not understood well yet [34]. Are we treating a disease by creating a new one and a worrisome experimental model for the development of Barrett’s esophagus and, eventually, esophageal adenocarcinoma? Or is the need for long-term PPI therapy the only consequence? At the moment, it is not possible to answer these questions, for several reasons. First, EA patients rarely spontaneously complain of reflux symptoms after treatment. Usually, they are so happy to have regained their eating capabilities that they tend to underestimate GERD symptoms eventually occurring after treatment. It is also possible that esophageal mucosa, chronically irritated and inflamed by the stasis of saliva and food, is less sensitive to the effect of gastric juice eventually refluxed. Moreover, in case of the appearance of GERD symptoms, the medical treatment with PPIs is extremely effective in controlling symptoms and, above all, esophagitis. If, for any reason, the medical treatment reveals to be insufficient or cannot be carried out in the long run (especially in young patients with long life expectancy), there will always be the possibility to perform a laparoscopic fundoplication sometime after a POEM [35]. Finally, even after effective treatment, the patients with EA should be per se endoscopically followed for the early diagnosis of a possible development of esophageal carcinoma [36], even if endoscopic surveillance is not recommended by current guidelines [37, 38]. This may occur after any treatment for EA, as well as in the untreated disease. It must be underlined, however, that the histologic type of cancer which develops in EA patients is nearly always squamous [36]. Even if reported in the literature, cases of development of Barrett’s esophagus or even adenocarcinoma in patients with EA are very rare [39]. The true relevance of the added effect of this iatrogenic gastroesophageal reflux in the development of complications and the risk of cancer for patients treated for EA is not well known yet. A case of early Barrett’s cancer has already been reported 4 years after POEM [40].

Our study has some limitations, though, some of them already mentioned. First, it is not an RCT, which still represents the gold standard in the evaluation of the effects of two or more different interventions or treatments. However, when RCT studies are not possible or not available for different reasons, PSM represents the probability of receiving treatment A rather than B for a patient with given observed baseline characteristics (potential confounders), by replacing these characteristics with a summary score, the PSM. This method has also been called a “quasi-randomization” method [19]. However, the data used for the PSM are still retrospective and non-randomized, even if prospectively collected. This can lead to a hidden bias due to latent variables that may remain after matching. Moreover, the strict statistical matching with PSM reduced our sample size by about half of the initial population in both groups. Second, we already stated that our results may not represent those achievable by centers with less experience with one or the other technique (i.e., the “real” world), since they were obtained by two centers of excellence in their respective treatments, thus probably offering the best possible results one can expect with both techniques, instead. Further, the two groups of patients had different median follow-up (longer in the LHD group), together with a different number of patients who reached the 5-year minimum follow-up (again, higher in the LHD group). There may be different reasons for this. We think that the patients who underwent an endoscopic procedure like POEM may give less importance to their treatment, somehow not bothering to show up at the scheduled visit or to respond to phone calls. On the other hand, patients who received proper surgical treatment probably realized the importance of their disease and the necessity to pursue regular follow-up better.

## Conclusion

In conclusion, our study confirmed that both POEM and LHD, if performed by experts in the procedures, represent two valid options for the treatment of EA at a minimum 5-year follow-up, with long-term outcomes well comparable between the two techniques. However, further comparative studies with even longer follow-up are certainly necessary to describe possible long-term differences of the two methods. In particular, they will surely clarify the real impact and long-term complications of iatrogenic GERD, undoubtedly higher for POEM compared with LHD. For these reasons, even though we do believe POEM is a good treatment option for EA and it should be alongside PD and LHD in the armamentarium of any referral center dealing with this rare disease, its claim to be considered the initial treatment for patients with EA, especially young patients, needs further, stronger evidence.
