# Correction: Five-year follow-up results of the Italian case–control study comparing poem and laparoscopic Heller myotomy using the propensity score

**DOI:** 10.1007/s00464-026-12989-x

**Published:** 2026-05-28

**Authors:** Andrea Costantini, Francesca Mangiola, Giovanni Capovilla, Rosario Landi, Luca Provenzano, Loredana Nicoletti, Michele Valmasoni, Cristiano Spada, Mario Costantini, Pietro Familiari, Renato Salvador

**Affiliations:** 1 Department of Surgical, Oncological and Gastroenterological Sciences, University of Padua, Padua, Italy; 2 Digestive Endoscopy Unit, Fondazione Policlinico Agostino Gemelli IRCCS, Università Cattolica del Sacro Cuore, Rome, Italy

**Correction to: Surgical Endoscopy** 10.1007/s00464-026-12910-6

The original online version of this article was revised to correct the presentation of the coauthors' names. The corrected names are:

Andrea Costantini, Francesca Mangiola, Giovanni Capovilla, Rosario Landi, Luca Provenzano, Loredana Nicoletti, Michele Valmasoni, Cristiano Spada, Mario Costantini, Pietro Familiari, Renato Salvador

The original article has been corrected.

